# Using multiple routine data sources linked to a trial cohort to establish the longer-term effectiveness of specialist home visiting in England: main results of the BB:2-6 study of the Family Nurse Partnership.

**DOI:** 10.23889/ijpds.v7i3.1829

**Published:** 2022-08-25

**Authors:** Michael Robling, Rebecca Cannings-John, Fiona Lugg-Widger

**Affiliations:** 1 Cardiff University

## Objectives

The Family Nurse Partnership (FNP) is a specialist home-visiting programme for first-time teenage mothers. Developed in the US, short-term outcomes (by age 2 years) were established in England in the Building Blocks trial. We used routine data (health, education, social care) linked to our trial cohort to assess longer-term impact.

## Approach

Mothers recruited to the trial and their first-born children were linked to health (Hospital Episode Statistics/HES: NHS Digital; Abortion statistics: Department of Health and Social Care), education/social care (National Pupil Database/NPD: Department for Education) data in England up to age 7-years. Analysis of data within in a trusted-research environment assessed programme impact upon child maltreatment, child development/educational and maternal life course outcomes when compared to usually provided health and social care support alone. Our primary outcome was child in need registration. Planned sub-group analysis included differential effects by maternal age, deprivation level, care experience and for child outcomes, sex.

## Results

Match rates for 1547 children were 97.4% (NPD) and 98.3% (HES). We found no difference between trial arms in proportion of children assessed as in need (adjusted odds ratio (aOR) OR:0.98, 95% confidence interval (CI): 0.74 to 1.31). Aside from a longer duration in care for children in the usual care arm (two months), there were no other differences in maltreatment outcomes. Children in the FNP arm were more likely to achieve a good level of development at reception age at age 4-5 years (aOR:1.24, 95%CI: 1.01 to 1.52) and, after adjusting for month of birth, to reach the expected standard in reading at Key Stage 1 at age around 7 years (aOR:1.26, 95%CI: 1.02 to 1.57).

## Conclusion

We found programme improvements for child development/educational achievement but not for child maltreatment outcomes. Additional sub-group analysis revealed some evidence of the programme benefiting mothers with greater baseline vulnerability and boys, consistent with previous trials. The study benefits from the linkage of administrative data to a previously randomised trial cohort.

